# Strain induced localization to delocalization transition on a Lieb photonic ribbon lattice

**DOI:** 10.1038/s41598-021-00967-3

**Published:** 2021-11-01

**Authors:** Diego Román-Cortés, Guillermo Fadic, Christofer Cid-Lara, Diego Guzmán-Silva, Bastián Real, Rodrigo A. Vicencio

**Affiliations:** 1 Departamento de Física and Millenium Institute for Research in Optics-MIRO, Facultad de Ciencias Físicas y Matemáticas, Universidad de Chile, Santiago, Chile; 2 Univ. Lille, CNRS, UMR 8523—PhLAM—Physique des Lasers Atomes et Molécules, 59000 Lille, France

**Keywords:** Photonic crystals, Micro-optics, Photonic devices, Electronic properties and materials

## Abstract

Ribbon lattices are kind of transition systems in between one and two dimensions, and their study is crucial to understand the origin of different emerging properties. In this work, we study a Lieb ribbon lattice and the localization–delocalization transition occurring due to a reduction of lattice distances (compression) and the corresponding flat band deformation. We observe how above a critical compression ratio the energy spreads out and propagates freely across the lattice, therefore transforming the system from being a kind of insulator into a conductor. We implement an experiment on a photonic platform and show an excellent agreement with the predicted phenomenology. Our findings suggest and prove experimentally the use of compression or mechanical deformation of lattices to switch the transport properties of a given system.

## Introduction

The understanding of transport and localization properties of different materials is the most relevant aspect in solid-state physics, not only from a fundamental point of view but also in terms of concrete applications. When synthesizing materials some deformations could emerge during the process; such as, compression, strain, defects and/or dislocations. However, they can also be added deliberately to enhance or to induce certain transport properties. In particular, compression and strain of materials have driven much attention lately because their transport properties can be dramatically modified. For instance, graphene can go from a semimetallic to an insulating phase when an uniaxial compression is applied^1,2^, phenomenon known as Lifshitz transition. In other two-dimensional (2D) materials similar transitions have been predicted. For example, black phosphorous switches from a semiconductor into a metal when it is subjected to an uniaxial strain^3^. This allows the control of the electronic transport properties on a nanodevice when an external electric field is applied^4^, which can be interpreted as a delocalization–localization transition. And, for germanium kagome lattices^5^ a transformation from a semimetallic into a semiconductor is observed when applying compression due to an increment of the orbital frustration that induces an electronic gap. Furthermore, the electronic structure and charge properties were studied in KCuSe and KCuTe^6^, finding that pressure effectively modifies the transport properties due to an enhancement of carrier mobility, which could have direct applications in optoelectronic technologies as, e.g., solar cells.

On the other hand, during the last decade, artificial lattices have arisen as feasible platforms to emulate and test most of the electronic properties predicted for solid-state-like materials^7–14^. Some of these systems have shown the ability to carefully engineer compression and, thus, exploring interesting phenomena that are sometimes unrealizable in natural and sinthetized materials. For example, the Lifshitz transition of graphene has been addressed using matter waves in optical lattices^15^, waveguides arrays^16^, arrays of microwave resonators^17^ and exciton-polariton lattices^18^. In the latter system, a predicted semi-Dirac scenario arises in graphene at a critical compression, which produces a highly anisotropic transport and particular localization features. Remarkable also, it has been experimentally shown in graphene photonic lattices that a smart design in term of compression or strain could induce a pseudomagnetic field, causing the rupture of Dirac cones and the appearance of Landau levels in the band structure^19–21^, which constitutes a clear delocalization–localization transition.

Besides compression properties, very fundamental condensed-matter phenomena has been experimentally proved in photonic lattices; e.g., Anderson localization^22^, topological insulation^23^, and Flat Band (FB) localization^24,25^. FB lattices have became an ideal solution for observing transport and localization phenomena on a completely periodic and linear configuration^26–28^, as well as for studies considering highly degenerated and interacting systems^29,30^. Moreover, during the last years, several contributions in optics have demonstrated different FB properties considering diverse lattice configurations^31–39^ and, thus, FB systems have emerged as a well-established and relevant research area where to continue asking/solving questions about the improvement or modification of fundamental properties in very different physical contexts; namely, electronic systems, magnetic lattices, metamaterials, mechanical lattices, quantum configurations, photonics, etc.^40–49^.

In this work, we explore the consequences of compression of a quasi-1D photonic lattice known as a Lieb photonic ribbon. Without compression, this lattice possesses four dispersive and one flat bands, and only nearest-neighbor (NN) couplings are relevant. When compression is applied, we observe that a next-nearest-neighbor (NNN) diagonal coupling starts to weakly affect the linear spectrum. It has been predicted that for Lieb-like lattices^50^ a weak diagonal coupling destroys the FB and all the spectrum becomes dispersive. However, in this work, we show that although diagonal coupling is not effectively zero, the FB phenomenology is still present and persists up to a critical compression value. We fabricate several dimer systems to fully characterize the coupling dependence and define a relation between coupling constants. Then, using this experimental information, we observe a localization–delocalization transition by theoretically analyzing the band spectrum as well as by numerically studying the transport through different compressed ribbon lattices. Afterwards, we fabricate several Lieb ribbon lattices using a femtosecond-laser written technique where we experimentally demonstrate this transition. We observe that, above a critical lattice distance (uncompressed ribbon), there is a tendency to localization, whereas below this distance (compressed lattice) the energy spreads out through the system inducing a delocalization transition.

## Femtosecond laser written Lieb photonic ribbon

**Figure 1:**
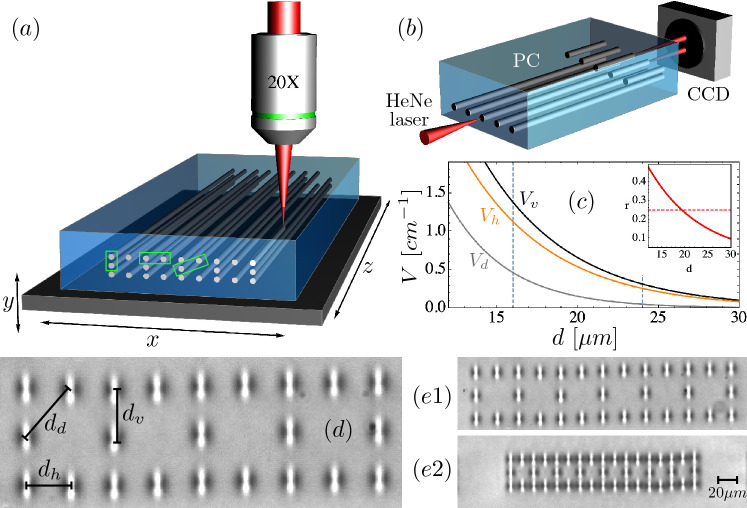
(**a**) Femtosecond waveguide writing setup. (**b**) Simplified characterization setup showing a focused HeNe laser beam at the input facet of a photonic chip (PC), and imaging onto a CCD camera. (**c**) Coupling *V* versus nominal distance *d* for vertical (black), horizontal (orange), and diagonal (gray) couplers. Vertical dashed lines are for $d=16$ and $24\ \upmu$m. Inset: fractional ratio *r* versus *d* (horizontal dashed line corresponds to $25\%$). (**d**) White light microscope zoom of a Lieb photonic ribbon. (**e1**) and (**e2**) Two ribbon examples with 33 and 43 waveguides for $d_v(d_h)=24(22.9)\ \upmu$m and $13.5(12.4)\ \upmu$m, respectively. This figure was drawn using Wolfram Mathematica 12, Flycapture2 and Omnigraffle 7.18.5.

Our aim is to study the effect of compressing a Lieb ribbon lattice and observe how the emergence of a diagonal NNN interaction $V_d$ affects the dynamics across the system. For this task is useful to understand first how the coupling constants are modified in our experimental platform. We fabricate several Lieb ribbon lattices by using a femtosecond (fs) writing technique^51^, as sketched in Fig. 1a. This fabrication method generates a small refractive index modification on a transparent glass-like material; in this case, a borosilicate Eagle XG wafer of width × length × thickness: $10\times 50\times 1$ mm (blue block in that figure). By focusing a Menlo BlueCut femtosecond laser (red beam in Fig. 1a), we are able to slightly modify the refractive index at the focal region, achieving a contrast $\Delta n\sim 10^{-4}-10^{-3}$. The axial geometry of the fabricated method produces vertical and elliptical elongated waveguides. Full waveguides are obtained by translating the glass wafer along the whole length $L=50$ mm, at a constant velocity of 0.4 mm/s. Three-dimensional control of the sample is achieved by a fully automatized Thorlabs micrometer stage (sketched as a dark plate in Fig. 1a), which allows us to translate the sample in *x*, *y* and *z* directions and, therefore, to generate arbitrary two-dimensional (*xy*) lattice configurations, with the *z* dimension acting as a time.

As a first step, we characterize the waveguide coupling dependence versus separation distance by fabricating sets of vertical, horizontal, and diagonal couplers (see dashed rectangles in Fig. 1a). These couplers consist of two waveguides separated by a variable center-to-center distance, where one waveguide has a full length ($L=50$ mm) and the other one a shorter length of 5 mm, as sketched in Fig. 1b inside the photonic chip (PC). We experimentally measure them by using a standard setup (see Fig. 1b), where a focused horizontally polarized HeNe laser beam excites a given waveguide at the input facet. Then, we obtain output light intensities on a CCD camera and extract the intensity information at every waveguide. The intensities follow a cosine-like dependence over propagation distance^51–53^, and they allow us to extract a coupling function for every waveguide separation. By compiling all the information, we obtain an exponential fit for a coupling versus distance dependence^51,52^, as shown in Fig. 1c, where $V_v(d_v)=26.43 \exp (-0.185 d_v)$ $\text {cm}^{-1}$ and $V_h(d_h)=21.41 \exp (-0.184 d_h)$ $\text {cm}^{-1}$. Due to the elliptical waveguide profile obtained from a fs-laser fabrication method^51,52^, vertical coupling $V_v$ (black line in figure) is always larger than the horizontal $V_h$ one (orange line in figure). However, we find a simple relation between these two coupling functions, obtaining that $V_v\approx V_h$ for $d_h=d_v-1.1\ \upmu$m, which is a very important detail for setting up a fabrication routine. Therefore, by adjusting $d_h$ and $d_v$ distances we are able to correct the anisotropy of the lattice, which implies having an effectively symmetric (square-like) lattice. As a consequence, for simplicity, we define a nominal distance *d*, as a control parameter in our simulations. Now, we characterize the diagonal coupling ($V_d$) considering a center-to-center distance $d_d=\sqrt{d_h^2+d_v^2}$. We immediately notice that the diagonal coupling (gray curve in Fig. 1c) is very small in comparison to NN (vertical and horizontal) coupling constants but, nevertheless, not strictly zero. We find that this diagonal NNN coupling has the following form: $V_d(d_d)=36.50 \exp (-0.273 d_d)$ $\text {cm}^{-1}$. In order to compare the magnitude of this NNN coupling constant, we plot a fractional ratio $r\equiv V_d/V_v$ over nominal distance *d* as inset in Fig. 1c. We notice that for $d \gtrsim 20\ \upmu$m, $V_d$ is less than $25\%$ of $V_v$.

After adjusting all coupling parameters, we start the fabrication of a total number of 14 photonic lattices. We split into two sets of arrays having a total number of 33 and 43 waveguides, for $d\geqslant 18\ \upmu$m and $d\leqslant 17\ \upmu$m respectively. Figure 1d shows a microscope image at the output facet of a fabricated photonic Lieb ribbon lattice, after white light illumination. Bright regions in this figure correspond to elliptical fs-written waveguides on a Lieb ribbon geometry, with relevant distances indicated explicitly at figure. This image shows dipole-like white light states^52^, which are originated due to the multiple wavelength excitation coming from a white light lamp. However, in this work, we will study our photonic lattices by using a red HeNe laser beam at 633 nm, for which all the waveguides are single-mode. Figure 1e shows two examples of different lattices at two regimes: uncompressed and compressed lattices.

## Model and linear spectrum

**Figure 2:**
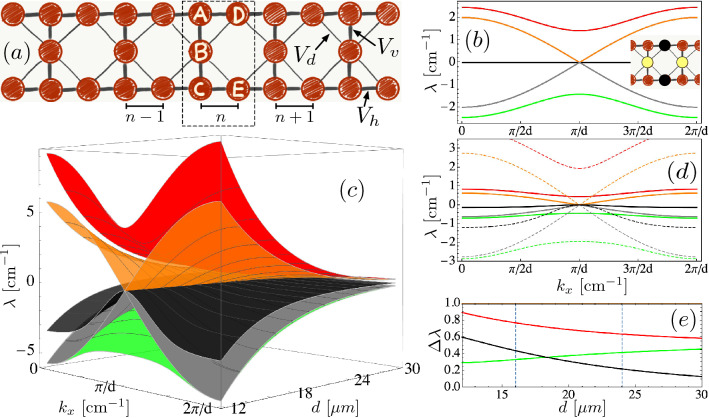
(**a**) A Lieb ribbon lattice with coupling coefficients indicated by arrows. Every filled disk represents an optical waveguide. (**b**) Linear spectrum versus transversal wavenumber $k_x$ for $V=1$ and $V_d=0$. Inset: flat band mode, where only yellow and black disks are different to zero. (**c**) Linear spectrum versus $k_x$ and nominal distance *d*. (**d**) Linear spectrum versus $k_x$ for $d=24\ \upmu$m and $d=16\ \upmu$m shown using full and dashed lines, respectively. (**e**) Band width $\Delta \lambda$ versus nominal distance *d*. This figure was drawn using Sketch 4.9.9, Wolfram Mathematica 12 and Omnigraffle 7.18.5.

In order to study a Lieb ribbon lattice, a singular flat band class^54^, we consider a tight-binding-like model with NN and NNN coupling constants due to an evanescent interaction in between close waveguides. The lattice structure is sketched in Fig. 2a, where the unit cell [see dashed rectangle] is composed of five sites: *A*, *B*, *C*, *D* and *E*. This lattice has three relevant coupling constants: horizontal $V_h$, vertical $V_v$, and diagonal $V_d$, as shown in this figure. Light dynamics is governed by a paraxial wave equation, which after applying coupled mode theory^26,55^ reads, in a general form, as

1 $$\begin{aligned} -i \frac{\partial u_{\vec {n}}}{\partial z}=\beta _{\vec {n}} u_{\vec {n}}+\sum _{\vec {m}} V_{{\vec {n}},{\vec {m}}} u_{\vec {m}}\ . \end{aligned}$$

Here, $u_{\vec {n}}$ describes the amplitude of a fundamental mode wave function located at lattice site $\vec {n}$, and *z* is the propagation coordinate. $\beta _{\vec {n}}$ corresponds to the propagation constant at site $\vec {n}$. For a homogeneous lattice, this parameter is equal for all waveguides and, without loss of generality, we simply set it as $\beta _{\vec {n}}=0$. $V_{{\vec {n}},{\vec {m}}}$ defines the evanescent coupling between sites $\vec {n}$ and $\vec {m}$, which naturally depends on the specific geometry and waveguides distance. Model (1) is generally referred as Discrete Linear Schrödinger (DLS) equations^26,55^, where *z* is the dynamical variable (time *t* in other contexts). On a solid-state scenario, $u_{\vec {n}}$ and $\beta _{\vec {n}}$ correspond to the wave-function of electrons and the site energy at lattice site $\vec {n}$, respectively, while $V_{{\vec {n}},{\vec {m}}}$ defines the tight-binding matrix coefficients.

As we described in previous section, we consider an effective symmetric lattice where $d_h=d_v=d$ and, therefore, we can assume $V_h=V_v=V$ in our model. We compute the linear spectrum of this ribbon lattice by assuming the following Bloch ansatz

2 $$\begin{aligned} \{A_n,B_n,C_n,D_n,E_n\}=\{A,B,C,D,E\}e^{i k_x dn}e^{i \lambda z}\ . \end{aligned}$$

Here, $k_x$ corresponds to the transverse wavenumber (Bloch wavevector), *d* to the nominal distance (lattice unit), and *n* to the discrete horizontal position through the lattice. As a Lieb ribbon lattice is classified as a quasi-1D system^26,31–34^, we have not included a vertical $k_y$ wavenumber due to the absence of transport on that specific direction. $\lambda$ represents the solution’s frequency or energy for linear modes (macromodes) of the full system.

First of all, by taking a null diagonal (NNN) coupling: $V_d=0$, the system converges to a standard 1D Lieb lattice, which is quite similar to a Stub^32,35^ or to a 2D Lieb^24,25,50,56^ system, but in this case it presents five linear bands instead of only three. In this limit ($V_d=0$), the system possesses a flat band (FB) located at $\lambda =0$, and four dispersive bands given by $\lambda (k_x)=\pm V\sqrt{2(1+\cos (k_x d))},\ \pm V\sqrt{2(2+\cos (k_x d))}$, where we have defined $V\equiv V_h=V_v$. Figure 2b shows a symmetric linear spectrum, where each positive frequency is paired to a negative one^57^. The flat band mode (see Fig. 2b-inset) has exactly the same profile than the one of a 2D Lieb lattice^24,25,56^, and it is formed by only four sites different to zero, with a staggered phase structure, and the following amplitude condition: $D=E=-V_v B/V_h$ and $A=C=0$. As this system has as much FB compact modes as closed rings along the lattice, all of them with equal propagation constant $\lambda =0$, they can be linearly combined to form spatially larger states^26,31^. For example, two neighbor FB modes constructively superposed form a spatial state having a larger peak at the central *B* site. Therefore, this localized state can be excited dynamically using a single *B*-site excitation, as we will numerically and experimentally show below, in the limit of a negligible diagonal coupling.

Now, we study the full case of considering a Lieb ribbon lattice with NN and NNN interactions as a more realistic model to understand the dynamics of this lattice, when considering the effect of compression. Along this work, we will assume that lattice compression implies a symmetric reduction of distances as the example shown in Fig. 1e. Therefore, we expect to switch on the diagonal coupling after a given critical compression, of course considering the realistic dependence of coupling constants described in Fig. 1c. For example, it is well known that a 2D Lieb lattice presenting NNN coupling ($V_d\ne 0$) losses its perfect flat band $\lambda =0$, but it nevertheless remains thin in comparison to the other two dispersive bands^50^. After inserting the plane-wave ansatz (2) in model (1), we obtain a set of five algebraic coupled equations. We solve the eigenvalue problem and find two analytical solutions $\lambda _{\pm }(k_x)=\pm V\sqrt{2(1+\cos (k_x d))}$. These bands are the same than in the previous perfect FB case ($V_d=0$) and are associated to transport along upper and lower rows of the lattice; i.e., a 1D-like transport with a total band width of 4*V* (the pairing of positive and negative bands^57^ is preserved on these two bands). The other three solutions can not be written in a compact form; therefore, we directly plot them in Fig. 2c as a function of $k_x$ and nominal distance *d*. As we described in the previous section, all couplings constants are a direct function of distance *d*: $V_h(d),\ V_v(d)$ and $V_d(d)$. Therefore, by varying *d* we are indeed modifying these coupling coefficients using the functionality shown in Fig. 1c, which was obtained directly from experiments. Figure 2c shows a strong modification of the linear spectrum in terms of distance *d*. We observe how for distances larger than $d\sim 18\ \upmu$m bands are quite flat, implying a slow transversal dynamics, due to the small band curvature^26,55^; however, strickly speaking, the spectrum is not flat: $\lambda \ne$ constant. In this regime, we expect a tendency to localization and weak dispersion due to the small available velocities in the system. On the other hand, for distances smaller than $d\sim 18\ \upmu$m, we observe a much broader linear spectrum, which grows rapidly, with larger slopes in general, something that guarantees a faster propagation through the lattice; i.e., a good transport regime (a wider spectrum implies larger available kinetic energies). Therefore, a lattice compression in real space produces a broadening in frequency space, as expected from reciprocal relations.

Figure 2d shows two band examples to illustrate the main differences observed in the linear spectrum. Full lines show the five linear bands for a distance $d=24\ \upmu \text {m}$, where we observe a narrow spectrum area and three bands which are notoriously flat/thin (red, black and green). This case corresponds to a relaxed (uncompressed) lattice and coupling constants are: $V=0.314$ $\text {cm}^{-1}$ and $V_d=0.052$ $\text {cm}^{-1}$ (i.e., $V\approx 6V_d$) being, therefore, a sort of realistic FB-like regime. Therefore, in this flat-band-like regime, we expect to observe a reduced transport^55^ when exciting the lattice edges (upper and lower rows), and a localization tendency when exciting the central row (*B* site). Dashed lines in Fig. 2d correspond to a distance $d=16\ \upmu \text {m}$; i.e., an already compressed lattice. We observe now that three bands are completely dispersive and broad (red, orange and gray), while two (black and green) are kind of mixed. They have regions where $\lambda \sim$ constant, which is kind of heritage from the previous FB-like limit. In order to analyze a bit more the bands properties, while compressing the lattice, we define a band width parameter as $\Delta \lambda \equiv [\lambda (k_x=0)-\lambda (k_x=\pi )]/|\lambda _{\pm }(k_x=0)|$. We compare all bands with respect to the total width of 1D-like bands as a reference, considering that these bands always produce transport and define a sort of dispersion scale in our lattice. Figure 2e shows $\Delta \lambda$ versus nominal distance *d*. By definition, $\Delta \lambda =1$ for orange ($\lambda _{+}$) and gray ($\lambda _{-}$) bands. We observe that the upper (red) band, although been always dispersive, has always a smaller band width than the 1D reference, in the interval shown in this figure. For larger distances, red and green band widths converge to a value $(\sqrt{3}-1)/\sqrt{2}\approx 0.5$ (as expected from Fig. 2b), while the black (central) band naturally converges to a FB with zero width ($\Delta \lambda \rightarrow 0$). For a decreasing distance *d*, black and red band widths increase and, therefore, we expect an increasing transport tendency. The lower (green) band width tends to saturate, showing the possibility of a weak tendency to localization as well. For $d\approx 18\ \upmu$m we observe a crossing region for an increasing black and decreasing green bands, which somehow could indicate a critical regime for observing a dynamical transition around this nominal distance. As the black band is originated at the FB for larger distances, when this band is not the thinnest one, we expect to observe a dominant transport across the system and, therefore, a localization–delocalization transition when compressing the lattice.

## Finite lattice dynamics

**Figure 3:**
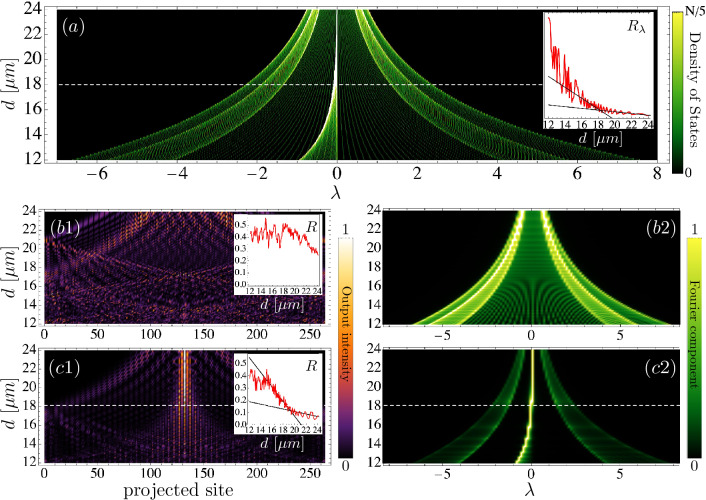
(**a**) Linear spectrum and density of states versus nominal distance *d*. Inset: dependence of the participation ratio in frequency space $R_{\lambda }$ versus distance *d*. (**b**) and (**c**) excitation of bulk-*A* and bulk-*B* sites, respectively. (**b1**) and (**c1**) intensity output profiles $|u_{\vec {n}}(z_{max})|^2$, for $z_{max}=100$, versus distance *d* where, for simplicity, the lattice is projected on a 1D row. Insets: participation ratio *R* for output intensities versus distance *d*. (**b2**) and (**c2**) dynamically excited frequency spectrum versus distance *d*. This figure was drawn using Wolfram Mathematica 12 and Omnigraffle 7.18.5.

Real systems are always finite and possess a fixed number of lattice sites *N*. We study numerically the properties of a finite system in order to obtain more realistic details for this quasi-1D photonic lattice. Although we are experimentally limited to the study of small systems only, due to the short available propagation distances of the fs waveguide writing technique, we study finite properties on a larger system having a total number of $N=263$ sites (which corresponds to a lattice with 52 closed rings). This choice is necessary to correctly analyze the properties of the linear spectrum of each lattice and determine more clearly the excited frequencies of the system as we will show below. First of all, we construct a tight-binding matrix $V_{{\vec {n}},{\vec {m}}}$ for lattices having different nominal distances *d*, which considers vertical, horizontal and diagonal coupling interactions only, as it was described in the second section considering the experimental data. We obtain the linear spectrum of every lattice system and calculate its density of states $D_{\lambda }(d)$, by computing an eigenfrequencies histogram, on a given interval and defined resolution. This allows us to not only see the projected linear spectrum for each lattice but also adding the information about the number of states associated to each frequency, as a way to predict the phenomenology of a given system in terms of the available states on each array. We show our results in Fig. 3a. For $d\gtrsim 18\ \upmu$m we notice that the spectrum is composed by a strong peak at $\lambda \approx 0$, with four dispersive bands clearly defined and isolated states in between. In Fig. 2c,d we observe that bands are always connected at $k_x=\pi /d$, with no gaps in between, been therefore a continuum of states in practice. The density is high close to central (originally flat) band and well disseminated in the dispersive surrounding bands. Therefore, we expect to observe a tendency to localization while exciting a bulk-*B* site, while dispersion and transport when exciting a bulk-*A* site. Below $d\approx 18\ \upmu$m we observe that the pseudo-FB peak starts broadening. In fact, at inset we show the participation ratio in frequency space $R_{\lambda }$ versus distance *d*, obtained by using the density of states information [$R_{\lambda }=R_{\lambda }(d)\equiv (\sum _{\lambda } |D_{\lambda }(d)|^2)^2/(\sum _{\lambda } |D_{\lambda }(d)|^4)$, where a small (large) value indicates few (many) strongly excited frequencies]. We observe how $R_{\lambda }$ clearly increases for distances $d\lesssim 18\ \upmu$m, indicating a noticeable change in the density of states for shorter distances *d* and compressed lattices. This naturally implies that the linear spectrum broadens and the FB phenomenology starts disappearing that is again a signature of a change in the lattice phenomenology.

We numerically integrate model (1) by exciting the lattice at a single-site. By exciting an *A*-site (or a *C*-site, due to lattice symmetry) we observe very good transport in Fig. 3b1, where we project the lattice sites intensities on a 1D row by applying a lattice ordering scheme with columns priority. In this figure, we chose a long propagation distance $z_{max}=100$ in order to observe a dynamically asymptotic regime as a consequence of well excited linear bands. Due to the large propagation distance, all the lattice is well excited and some fast waves are reflected at lattice surfaces. The participation ratio *R* shown as inset in this figure indicates a rather constant dissemination of the energy, with values larger than 0.2 for different distances *d* (an *R* value close to zero corresponds to a localized profile, while $R\sim 1$ indicates a delocalized one^26,55^). The dynamically excited spectrum in this case is shown in Fig. 3b2. This is obtained by Fourier transforming the wave amplitude at every lattice site, in the interval $z\in \{0,z_{max}\}$. This gives us the frequencies excited on a specific lattice position, which are then numerically integrated by simply summing the absolute value over the whole lattice^58^. This gives us the excited spectrum in the dynamics and the key modes which are responsible for the observed spatial profiles, where we observe quite clearly that dispersive (surrounding) bands are excited the most, with an almost absent central (flat-band-like) band. On the other hand, Fig. 3c1 shows the compiled results for a *B*-site excitation. We clearly observe a dynamical transition at $d\approx 18\ \upmu$m (see dashed horizontal line), from a localized/trapped output profile into a completely broad/dispersed spatial pattern. We include an inset showing the participation ratio *R* versus distance *d* computed with the data shown in this figure. We observe a dynamical transition around $d\approx 19\ \upmu$m (see linear fits included), with a notorious change in the slope as an indication of a larger excited area after this transition. Therefore, in this asymptotic regime, we clearly predict a localization–delocalization transition when compressing the Lieb ribbon lattice, below a critical distance *d*. Figure 3c2 shows the frequency spectrum excited during the propagation described in Fig. 3c1. Figure 3c2 shows quite clearly the persistence of the original flat (central) band located at $\lambda \approx 0$, when exciting a *B*-site, where broader (surrounding) dispersive bands are only weakly excited in this case. This central band is strongly excited and its width properties will somehow define the observed transition, what from the presented data would occur for a band width $\Delta \lambda \approx 0.35$, as shown in Fig. 2e. Then, for $d\lesssim 18\ \upmu$m we see how this main peak broadens and shifts to smaller frequencies, and spatial output profiles increase abruptly, as a result of an increasing band width.

These results show that a localization–delocalization transition, appearing when comprising the lattice with the consequent reduction of nominal distance *d*, is originated due to the persistence of localization properties of the central band of this lattice, which is responsible for the existence of a FB when the diagonal interaction becomes negligible. Therefore, we theoretically and numerically predict that FB properties will not disappear immediately when switching on the diagonal interaction and that will persist up to a given threshold distance, which we have found is in the range of $d\in \{18,20\}\ \upmu$m, for the experimental parameters we are using in this study. From a more theoretical perspective, and using the previous analysis, we could claim that this transition would happen when band widths are all larger than $\Delta \lambda \approx 0.3$, for this specific Lieb ribbon lattice. Nevertheless, the transition would be observed only if the right site is excited. As the input site directly defines the specific bands excited dynamically, there will be some sites showing the localization–delocalization transition and other sites showing only transport.

## Experimental results

**Figure 4:**
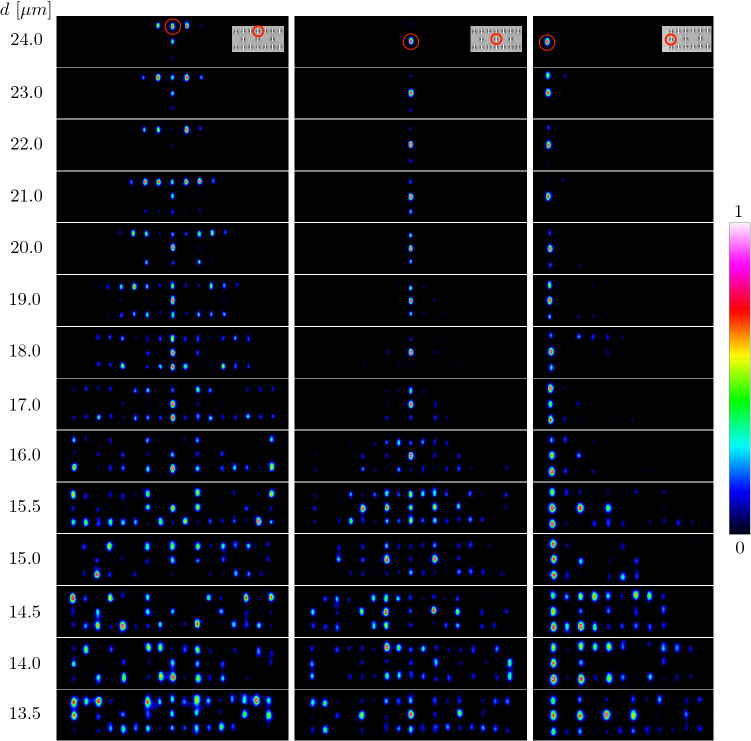
Left, center and right columns show the experimental output intensity pattern for *A*-bulk, *B*-bulk, and *B*-edge input excitations, as indicated at insets by red circles. Nominal distance *d* decreases downwards, as indicated directly beside left column. The aspect ratio was corrected to compare different lattices on a similar visual scale. A rainbow-like color scale is applied, which is normalized to the peak power of every image. This figure was drawn using Thorlabs’ Beam 8.0 and Omnigraffle 7.18.5.

As commented in the second section, we fabricated 14 ribbon lattices to experimentally study the localization–delocalization transition produced by a symmetric lattice compression. By using a characterization setup as the one shown in Fig. 1b, we are able to study the dynamics for all the fabricated lattices and measure the intensity output pattern after a propagation distance of $L=50\,\text {mm}$. In Fig. 4 left-column we observe the excitation of an *A*-bulk site, as shown in inset (this is equivalent to a *C*-site excitation as well). We clearly observe a good transport scenario when exciting this site. The energy spreads out homogeneously across the lattice, due to the fact that only dispersive bands are excited. For lattices with a larger nominal distance *d*, the diffraction area is narrower due to the smaller maximum velocities excited (narrow linear spectrum as shown in Fig. 2c). Therefore, the necessary propagation distance for observing a whole energy dissemination would be much larger than 50 mm, as described numerically in Fig. 3b1. For $d\geqslant 18\ \upmu$m we observe that in the opposite (bottom) row already light is diffracting in a 1D-like form, although light has not arrived to the edges strongly. This is the main reason for fabricating smaller lattices up to this nominal distance, as transport is still occurring slowly. Then, for $d\leqslant 17\ \upmu$m we increased the system size to a total of 43 waveguides and immediately observe how light spreads through a larger transversal area. We notice that for an even shorter distance *d* light explores quite well the whole lattice with noticeable reflections at edges as a clear manifestation of a broader linear excited spectrum and larger kinetic energies. Therefore, we have shown quite clearly the transport regime for these ribbon lattices, that is independent of the nominal distance *d* but, naturally, it depends on the dynamical coordinate *z*.

Figure 4 center-column shows the excitation of a *B*-bulk site for Lieb ribbon lattices, while symmetrically compressing the system and, effectively, reducing the nominal distance *d*. We clearly observe that for $d\geqslant 18\ \upmu$m the energy remains completely localized at the input region, independent of a distance *d* reduction, which for an *A*-site excitation already shows a very good transport. The energy remains localized mostly at the input site, therefore being a quite localized profile with a participation ratio of $R\sim 1$, which for finite and infinite systems corresponds to a perfect localization. This localization tendency comes from the FB heritage and shows very clearly the preservation of FB properties although, strictly speaking, diagonal coupling $V_d\ne 0$ and, consequently, the FB compact states are not system eigenmodes. Then, for smaller distances the energy starts to weakly spread through the lattice. We observe for $d=15.5\ \upmu$m that the maximum intensity is not at the *B* input site anymore; as a result, the transition into delocalization has started to occur quite clearly. Finally, for smaller distances the spectrum is even wider and the FB heritage is simply lost. The energy is well disseminated across the lattice as an effect of compressing the system. Therefore, we have observed a localization–delocalization transition experimentally and showing quite clearly the possibility of changing the transport properties abruptly by comprissing the lattice.

As an extension, we experimentally excited the surface of every lattice to also show the transition under compression at the lattice edge. We injected light at the input facet of a *B*-site as shown by inset in Fig. 4 right-column. Again, we observe high localization up to a nominal distance of around $d=18\ \upmu$m, with a single-peaked profile. For $d\leqslant 17\ \upmu$m we observe a spatial transition and observe that the profile has now two main peaks, although it is still quite localized and compact, but showing that a delocalization transition is starting to occur also at the lattice edge. For even smaller distances, we observe how the energy spreads out into the system and how the dispersive nature of the compressed lattice manifests. Therefore, a relaxed Lieb ribbon lattice behaves as an insulator, in the sense of not conducting energy through the system. Under compression, the energy is able to spread out across the lattice and the system becomes a conductor-like media.

## Conclusions

In conclusion, we have studied theoretically and experimentally a localization–delocalization transition induced by strain in Lieb ribbon lattices. We found the analytical solutions of the system and how they are affected by a compression value, which in photonic lattices can be understood as the inclusion of next-nearest-neighbor coupling. For small values of compression, the solutions are localized, and the system presents a low transport. But under severe compression, the solutions are extended, and a high transport is obtained. This transition from localized to delocalized states was observed by using excitations in *B* sites of the lattice, where properties of localized states, reminiscence of flat band states, prevails until a critical value of coupling. These results show that Lieb lattices, and presumably other lattices possessing flat bands, are good candidates to the study of band structures modification and the tuning of transport properties through compression.

## Methods

### Sample fabrication

The photonic lattice used in our experiment was fabricated using the femtosecond laser writing technique^51^. By focusing a laser beam on a borosilicate wafer, we are able to locally modify the refractive index. Then, we translate the sample at fixed velocity and create a complete waveguide inside the glass plate. Depending on the transversal pattern of the specific lattice, we repeat this procedure on several positions and fabricate a full photonic system.

## Data Availability

The datasets generated during and/or analyzed during the current study are available from the corresponding author on reasonable request.
